# The Stemness Phenotype Model

**DOI:** 10.5402/2012/392647

**Published:** 2012-08-08

**Authors:** M. H. Cruz, Å. Sidén, G. M. Calaf, Z. M. Delwar, J. S. Yakisich

**Affiliations:** ^1^Department of Clinical Neuroscience R54, Karolinska University Hospital, Karolinska Institute, Stockholm, Sweden; ^2^Department of Neurology, Karolinska University Hospital, Stockholm, Sweden; ^3^Instituto de Alta Investigación, Universidad de Tarapacá, Arica, Chile; ^4^Center for Radiological Research, Columbia University Medical Center, New York, NY, USA

## Abstract

The identification of a fraction of cancer stem cells (CSCs) associated with resistance to chemotherapy in most solid tumors leads to the dogma that eliminating this fraction will cure cancer. Experimental data has challenged this simplistic and optimistic model. Opposite to the classical cancer stem cell model, we introduced the stemness phenotype model (SPM), which proposed that all glioma cells possess stem cell properties and that the stemness is modulated by the microenvironment. A key prediction of the SPM is that to cure gliomas all gliomas cells (CSCs and non-CSCs) should be eliminated at once. Other theories closely resembling the SPM and its predictions have recently been proposed, suggesting that the SPM may be a useful model for other type of tumors. Here, we review data from other tumors that strongly support the concepts of the SPM applied to gliomas. We include data related to: (1) the presence of a rare but constant fraction of CSCs in established cancer cell lines, (2) the clonal origin of cancer, (3) the symmetrical division, (4) the ability of “non-CSCs” to generate “CSCs,” and (5) the effect of the microenvironment on cancer stemness. The aforenamed issues that decisively supported the SPM proposed for gliomas can also be applied to breast, lung, prostate cancer, and melanoma and perhaps other tumors in general. If the glioma SPM is correct and can be extrapolated to other types of cancer, it will have profound implications in the development of novel modalities for cancer treatment.

## 1. Introduction

The identification of putative cancer stem cells (CSCs) in tumors some years ago gave rise to new concepts in cancer biology, and consequently new dogmas in the cancer field were established. The classical cancer stem cells model (CSM) proposes that all cancer types have a subpopulation of cancer stem cell responsible for resistance to chemo- and/or radiotherapy, concluding that eliminating this subpopulation of CSCs will cure cancer [1–5]. However, there is no consensus among experimental data regarding key issues that are important for the establishment of effective treatments. For instance, the percentage of cancer stem cells detected in glioma cell lines tumors varies from less than 1% to 100% (for review see [6]). The differences have also been observed in other types of cancer (see below). However, these discrepancies, which might be well due to differences in methodology and criteria used to detect and characterize these cells have important clinical consequences. If the percentage of CSCs is rare (<1%), the elimination (if feasible) of this fraction with some kind of targeted treatment would indeed be a success, providing that non-cancer stem cells (non-CSCs) are easily controlled by other cytotoxic or cytostatic therapies. In the other extreme scenario, where 100% of cancer cells are CSCs, the effective therapy will require a novel treatment able to eliminate 100% of cancer cells at once in order to prevent regrowth. 

Based on our observations of proliferation kinetics of mixed cell cultures, we have developed a novel model of glioma biology (Stemness Phenotype Model, SPM), which proposes that all glioma cells have the potential to develop stem cell properties and that the stemness degree depends on the microenvironment [6]. Although the SPM was almost entirely derived from experimental data obtained from cell lines, it is important to keep in mind that the recent interest in the cancer stem theory comes after the isolation of putative cancer stem cells from a variety of well-established cell lines. More important, the tools and criteria to isolate and/or identify putative cancer stem cells (e.g., stem cell markers, neurosphere, clonogenicity) are similar in both stable cell lines or freshly isolated primary cancer cells. In general, the criteria to define CSCs are (1) extensive self-renewal ability, (2) cancer-initiating ability on orthotopic implantation, (3) karyotypic or genetic alterations, (4) aberrant differentiation properties, (5) capacity to generate non-tumorigenic end cells, and (6) multilineage differentiation capacity [7, 8]. Experimental data from primary cells cultured under stem cell propagating conditions that are more relevant than cell lines are also included in this paper (see examples in Tables 2 and 3) and further support the SPM. During the last two years, this idea that a stem cancer cell may not have a unique state of stemness has also been expressed by others. Thus, in a recent paper, Hatiboglu et al. wrote: *“…This raises the possibility that “stemness” is a dynamic property that many glioma cells may potentially adopt, depending on circumstance. If this is true, targeting gCSCs *(glioma Cancer Stem Cells)* in isolation should not be considered a panacea for GBM, since even after successful eradication of gCSCs, other glioma cells may acquire gCSC properties and reconstitute a population of gCSCs*.” [9] reinforcing the notion by Dong and Huang that “*simply eradicating the existing GSCs* (Glioma Stem Cells)* is not enough to be a cure for gliomas*” [10]. On the other hand, recently published observations are in agreement with the SPM, (1) the radioresistance of glioma cells has been attributed to a putative “microenvironment-stem cell unit” giving less importance to the intrinsic characteristic of glioma stem cells [11]. Jamal et al. found that the brain microenvironment preferentially enhances the radioresistance of glioma stem-like cells [12], (2) C6 glioma cells growing under different culture conditions showed distinct stem cell properties and were tumorigenic as predicted by the SPM [13], (3) similar to the C6 glioma cell line, most of the human glioma SHG44 cells could be considered gCSCs [14], and (4) the capacity of cancer progenitor cells to dedifferentiate and acquire a stem-like phenotype supports a bidirectional conversion [15]. 

**Figure 1:**
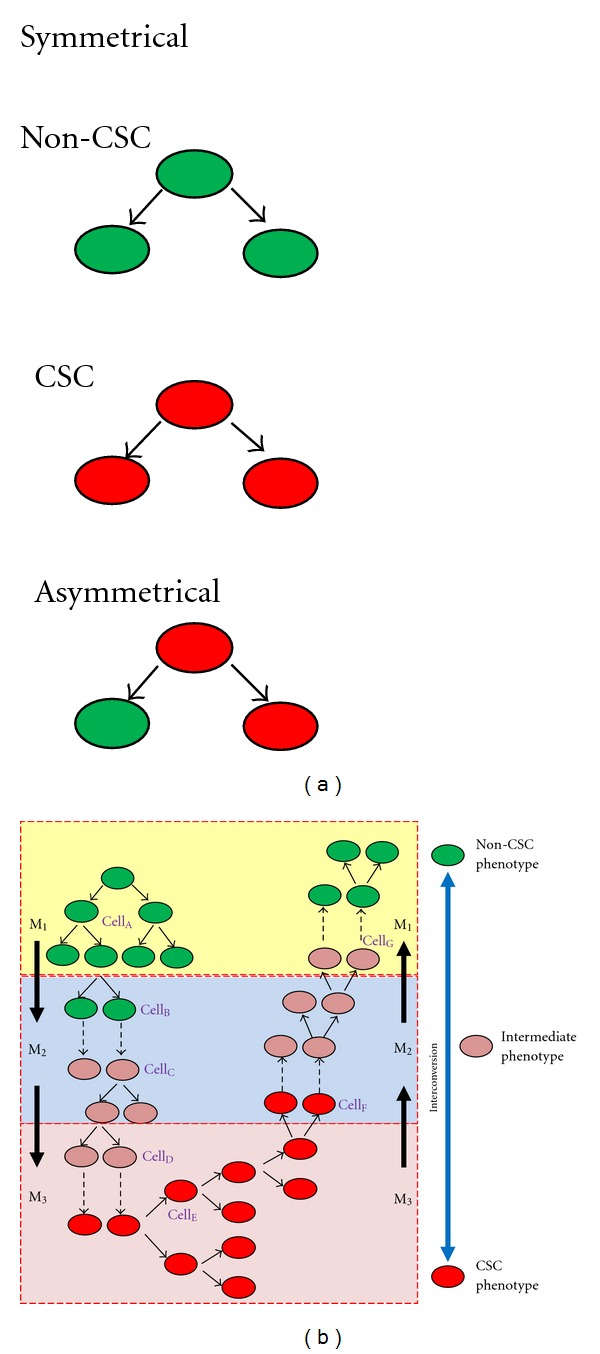
The stemness phenotype model proposes that all cancer cells have stem cell properties and that the stemness of individual cell depends on the microenvironment. (a) Cell division mode. (b) Schematic representation of the dynamic of cancer cells within a tumor. For simplicity, the depicted tumor is composed of three compartments (microenvironments M_1_, M_2_, and M_3_), and only three cell phenotypes are shown (non-CSC, intermediate, and CSC phenotype). According to the SPM, (1) all cancer cells (non-CSCs and CSCs) divide symmetrically, (2) changes in the microenvironment modify the phenotype of individual cells (e.g., broken arrows in M_1_→M_2_ or M_2_→M_3_, indicate phenotype transition and not cell division), thus, (3) non-CSCs are able to generate CSCs when changes in the microenvironment favours this conversion (e.g., from M_1_→M_2_→M_3_), (4) isolated single cells can generate a tumor (or a cell culture) containing different cell phenotypes. For instance, if a single cell (e.g., Cell_A_ or Cell_B_) is transferred alone to a microenvironment that allows its survival (e.g., M_2_) this cell has the potential, by only symmetrical division, to generate cells with intermediate phenotype as wells as cells with stem cell phenotype (clonal origin of tumors and cell lines). The same can be predicted for other cells regardless of their phenotype (e.g., Cell_C_–Cell_G_), and (5) the model predicts that the relative percentage of CSCs in a given tumor will depend on the microenvironmental profile of each individual tumor (e.g., expanding the size of the M_3_ compartment will result in an increase number of cells having a pure CSC phenotype). In this figure non-CSC (especially in Figure 1(b)) refers to any cancer cell that does not show any trait of stemness and not to a normal nontransformed cell.

The aim of this short review is to expand the concept of the stemness phenotype model to other cancer types. Five aspects of cancer biology supporting the stemness phenotype model for other tumors will be reviewed and discussed: (1) the presence of a rare but constant fraction of stem cell subpopulation in established cancer cell lines, (2) the clonal origin of cancer, (3) the symmetrical division, (4) the ability of “non-cancer stem cells” to generate “cancer stem cells,” and (5) the effect of the microenvironment on cancer cell phenotype.  Figure 1 provides an integrative view of these five aspects of cancer biology. 

## 2. Presence of a Rare but Constant**** Fraction of Stem Cell Subpopulation**** in Established Cancer Cell Lines 

The presence of cancer cells with stem cell properties has been reported in several cancer types. In this paper, we show examples for breast cancer, lung cancer, prostate cancer, and melanoma (Tables 1–4) and also from other tumors where crucial experimental data have been reported. CSCs or cancer stem-like cells (CS-LCs) have also been identified in colon [38], hepatic [39], pancreatic [40], thyroid [41], bladder [42], cervix [43], ovarian [44] cancer, urothelial cell carcinomas [45], renal carcinomas [46], chordomas [47], and, in general, in all types of tumors where these cells had been searched for. The existence of a rare but constant fraction of CSC in established cell lines can easily be explained if one assumes that CSC and non-CSC have exactly the same population doubling time. In this case, if a cell line contained 1% of CSC and 99% of non-CSC, this proportion should be preserved no matter how many passages the cell line underwent through the years. For instance, the C6 glioma cell line [48] and the A549 human lung cancer cell line have been propagated *in vitro* thousands of times [49]. However, one of the key characteristic of CSCs often highlighted in the stem cell theory is the quiescent slow-cycling phenotype [50], that is, CSCs often divide slower than non-CSCs but the opposite also exists (see below). As previously discussed for the glioma C6 cell line [6], it is unlikely that a cell line containing a mixture of slow and fast proliferating cells could maintain a constant fraction of stem cells since, after continuous passage, the faster subpopulation should overgrow the slower subpopulation(s) as we clearly showed in the original SPM article (see Table  2 in [6]). In the human bladder transitional cell cancer cell line T24, originally established in 1973 [51], the SP cells comprised approximately 34.7% of the total cells [52]. Contrary to putative glioma stem cells that grew slower than nonstem cells [7], the SP cells isolated from the T24 cell line grew significantly faster (more than twice) than non-SP cells [52]. In this case, after certain number of passages, this cell line should contain only SP cells. Perhaps HeLa cells, the first human cancer cell line grown in culture, represents one of the most extreme cases. The HeLa cell line was established in 1951 [53] and extensively propagated since then in many laboratories and still at present contains a subpopulation (1.1 ± 0.19% by the side population method) of self-renewing, highly tumorigenic, cancer initiating cells [43]. Cancer stem-like cells have also been isolated from HeLa cells by the sphere culture method [43, 54]. 

To explain the occurrence of subpopulations of cells with different growth rate is even more problematic in the case of the MCF-7 cell line. In one study, six different subpopulations were isolated by Percoll gradient centrifugation. Each of these subpopulations showed different growth rate showing increase ratio of 1.1x, 3.8x, 6.2x, 6.8x, 7x, and 16x between the cell numbers at day 8 and at day 0 [55]. In this study, the authors suggested that the fastest fraction contained the stem cells. If the MCF-7 cell line was composed of six different and independent subpopulations, after repeated passages should it be composed mostly of the stem cells. This is not likely the case. 

 Three subpopulations of cancer cells having different tumorigenicity have been reported for the established HC116 cell line [56]. In these cells, a small but not significant difference in the population doubling time (PDT) was observed. However, even small differences in the PDT are enough to enrich one population [6]. In melanomas, it has been reported that (1) three stable metastatic cell lines (in spite of constant passage for over a year) maintained a heterogeneous population of cells with different morphologies including small ovoid, spindle, flat polygonal, and large dendritic forms being the larger cells that proliferated faster. The purified single tumor cells retained the capacity to give rise to heterogeneous cultures [57], (2) a small subpopulation of slow-cycling cells (JARID1B+) was present in *in vivo* and *in vitro* studies. This fraction had doubling times of >4 weeks when compared to the rapidly proliferating main population (48 hs). Remarkably, JARID1B− cells were found to be able to generate the JARID1B+ subpopulation [58]. In these cases, the ability of single melanoma cells or specific subpopulations to regenerate a heterogeneous culture explains the maintenance of the tumor heterogeneity (see below Section 2.3). 

Ware et al. reported the isolation of subclones from the parental lines of human breast (MCF-7 and T-47D), prostate (DU145 and PC-3), lung (A427 and A549), colon (HCT-116 and HT-29), and bladder (TCCSUP andT24) cancer cells. All these subclones showed different PDT. For instance, while the parental T-47D cells had a PDT of 55.3 hrs the eleven isolated subclones showed PTD between 40.5 and 65.4 hrs. Similar results were observed in all other subclones isolated from the parental cell line [59]. Although the subclones were not characterized in terms of stemness, the great variability in the PDT suggests that each subclone corresponds to a specific phenotype and that the subpopulations having the longest PDT should be constantly generated. 

In conclusion, this type of analysis strongly suggests that cells with specific phenotype (e.g., stem cell phenotype in the glioma cell lines or SP cells in the T24 bladder cell lines) should be constantly generated in order to not disappear after continuous passages.

### 2.1. The Clonal Origin

One of the key proposals of the SPM is that all cancer cells phenotypes must be originated from one single parent cell and that tumor heterogeneity is generated afterwards from that single cell. It is important to clarify that in this paper the discussion about the clonal origin is restricted to the tumor heterogeneity found in cell lines and tumors and not to the origin of cancer. Early studies provided evidence for a clonal origin of teratocarcinoma [60] and CML [61]. The clonal origin has also been documented for the glioma C6 cell line [62] and is likely to be the case for the A549 human lung cell line [49]. Although other cell lines were not isolated from single cells, a careful analysis of the data suggests that the different cell lines present in cell cultures might have been originated by clonal evolution. Thus, the above-mentioned MCF-7 cell line (with its 6 different subpopulations isolated by Percoll gradient representing six different original and independent clones) was likely of clonal origin [63] since, as the authors noticed, two fractions were able to regenerate the different subpopulations [55] clearly suggesting that they represented different stages of differentiation. In another study, 5 clones and subclones were isolated after long-term passage from a single specimen of a human hepatocarcinoma. These clones showed different PDT, drug resistance, expression of stem cell markers, and tumorigenic potential. However, genomic analysis indicated a possible clonal evolution [64]. 

Studies on tumor evolution by sequencing DNA isolated from tumor tissue suggested a clonal origin [86, 87]. This finding was later supported by DNA sequencing of single cells [88]. These data support also the notion that during malignant transformation the primordial cancer cell has or acquires stem cell properties and as a result, the ability to form heterogeneous tumor. It appears that the primordial cancer cell originates a tumor that grows and invades normal tissue, giving origin to different microenvironments in which the stemness of the daughter cells changes accordingly. 

 A model where a CSC and a non-CSC are originated independently is very unlikely to occur since the probability that two cells become malignant (one as CSC and another as non-CSCs) at the same time and place should be very low. We want to point out that the exact origin of a cancer cell, from either a stem cell precursor or from a differentiated cell that acquired stem cell properties, is a different issue and the SPM does neither support nor refute any of them. However, a clonal origin of cancer (as suggested by some of the studies above mentioned), if ever confirmed, will be in better agreement with the SPM. 

### 2.2. The Symmetrical Cell Division

Cells that divide symmetrically will always produce identical daughter cells. In contrast, a CSC that divides asymmetrically produces (a) one daughter CSC that can again divide asymmetrically and (b) one daughter non-CSC that is terminally differentiated and cannot produce new CSCs (Figure 1(a)). To explain the experimental finding that all cancer cells have stemness properties, the SPM predicts that all or most cancer cells should divide symmetrically. Indeed, a recent paper concluded that the generation of cellular diversity in glioma occurs mainly through symmetric cell division [89]. In oligodendrogliomas, the loss of asymmetric cell division has been associated with malignant transformation of oligodendrocyte precursor cells (OPCs). While OPCs divide asymmetrically, oligodendroglioma cells divide symmetrically [90]. The SPM model shows in Figure 1(b) that symmetrical division of cancer cells concomitant with microenvironmental-dependent phenotype changes is enough to explain the heterogeneity of tumors and the ability of each cancer cell to generate a new tumor. 

### 2.3. Ability of “Non-Cancer Stem Cells” to Generate “Cancer Stem Cells” (Interconversion)

In contrast to the classical CSC model and according to what we have been discussing so far, the SPM proposed that CSCs and non-CSCs can interconvert into each other. The more important consequence of this event is that cells labelled as non-CSCs can generate CSCs that ultimately induce a new tumor. Experimental data obtained from a variety of tumors support this hypothesis for instance (1) clonal analysis of prostate cancer cells showed that some CD44^−^ Du145 cells (100% purity) could give rise to CD44^+^ cells in culture [35], (2) in the MCF-7 breast cancer cell line, the non-SP cells that were recultured during 7 days after being sorted indeed contained SP cells indicating that the non-SP fraction gave rise to a new SP subpopulation [22], (3) in melanoma (see also above), both ABCB5+ and ABCB5− as well as CD133+ and CD133− cells were able to form tumors that exhibited similar heterogeneity regarding CD133 expression. Similar results were observed with a panel of other surface markers including CD133, CD166, L1- CAM, and CD49f (for details, see [91]). The authors concluded that “*These data suggest a phenotypic plasticity model in which phenotypic heterogeneity is driven largely by reversible changes within lineages of tumorigenic cells rather than by irreversible epigenetic or genetic changes*” [91], (4) a direct conversion from a non-CSC phenotype to a CSC phenotype was demonstrated in breast cancer cells: exposure to conditioned media stimulates non-CSCs to become CSCs, and IL6 was enough to drive this conversion in genetically different breast cell lines, human breast tumors, and a prostate cell line [92], and (5) Gupta et al. [93–95] experimentally demonstrated interconversion (and determined the transition probabilities) of subpopulations for two breast cancer lines that were purified for a given phenotype [93–95]. The comparison between our predictive model (see Figure 2 II in Cruz et al., [6]) and experimental results (see Figure  2D in Gupta et al., [93–95]) shows striking similarities. 

If we consider that the PDT of non-GSCs and GSCs are approximately 28–30 h, and 55–60 h, respectively, then GSCs should be constantly generated from non-GSCs. In contrast if we consider the PDT of T24 bladder cell lines where the PDT of the non-SP fraction is more than twice longer than the SP fraction that is associated with cancer stem cells [52], then non-SP cells should be constantly generated from SP cells. These two opposite cases further support the idea that the interconversion between cancer cell phenotypes actually takes place and can be in any direction and explains the finding that isolated single cells can repopulate the original tumor as reported for C6 glioma cells [96, 97].

### 2.4. Effect of the Microenvironment on Cancer Cell Phenotype (Modulation of Stemness by External Factors)

According to the SPM, cancer cells have a high degree of plasticity and the phenotype displayed by them is highly modulated by the microenvironment. Several external factors have indeed been shown to modulate the stemness of several types of cancer cells (Table 5). The tumorigenic capacity of malignant ovarian ascites-derived cancer cell subpopulations has been shown to be niche-dependent [98] and subpopulations derived from a single tumor of ovarian clear cell carcinoma that were clonally expanded showed intratumoral phenotypic heterogeneity highly dependent on the tumor microenvironment [99]. For prostate cells, the ability to express a highly or scarcely aggressive phenotype was shown to be dependent on factors released from the tumor environment [81]. Other external factors such as bile and inflammatory mediators were shown to activate stem cells in Barret's metaplasia, a risk factor for esophageal adenocarcinoma [100, 101]. 

## 3. Clinical Implications

It is becoming more evident that the simplistic established classical CSC model and its corresponding dogmas for cancer biology are in a need of urgent revision. A clear comprehension of cancer stem cell biology is obviously essential for future medical research ranging from preclinical drug testing of anticancer drugs to advanced clinical trials. Other models attempting to explain the controversy between experimental findings and the predictions of the classical stem cell models have been recently proposed, among them the “complex system model” [84], the “Dynamic CSC model” [85], and the “Dedifferentiation”model [15]. Similar to our SPM, those models deviate from the classical CSC model and propose or predict that (1) all cancer cells are potentially tumorigenic. Therefore, interconversion between phenotypes is implicit in these models and (2) all cancer cells should be the therapeutic target. Table 6 shows the similarities and differences between the classical CSC models, the SPM, and these other alternative models of CSCs. The main difference between the SPM and other CSCs models is that in the SPM, the origin of all cancer cell subpopulations can be entirely attributed to microenvironment-driven phenotypic changes without additional mechanism (e.g., genetic mutations) (Figure 1). Thus, in the SPM “stemness” is an inherent property of all cancer cells. This likely to be the case for the dynamic CSC model that is one of the new alternative models that closely resembles the SPM. In the dynamic CSC model, CSCs and differentiated cell can interconvert to each other depending on signals from the microenvironment shaped by stromal cells (myofibroblasts, endothelial cells, mesenchymal stem cells, or infiltrating immune cells) [85]. In other systems such as the complex system model, several tumor-initiating cell types may coexist due to genetic and epigenetic changes within a single tumor. While genetic mutations produce new tumor cell, epigenetic changes may produce progeny that can temporarily adopt therapy resistance and expression of different cell markers [84]. In the reprogramming model, CSCs and relatively differentiated cells coexist and undergo bidirectional conversion. For instance, cancer progenitor cells are capable of dedifferentiation into a stem-like phenotype due to either genetic mutations or microenvironmental signals [15]. All these novel models of CSCs (including the SPM) may complement each other and even coexist in a complex heterogeneous tumor. For instance, in addition to intratumoral microenvironmental phenotypic-driven heterogeneity, genetic mutations (likely to occur due to genomic instability of cancer cells) may create new subpopulations of CSCs in the same tumor. If the glioma SPM is accurate and can be extrapolated to other cancer types, the vast majority of therapies aimed to eliminate one or few cancer stem cell subpopulations (e.g., CD133+ cells) will be a waste of time, resources and ultimately cost more lives. Even in the possible cases that (1) only a subset of non-CSC can interconvert to CSC rather than “all tumor cells” or (2) tumors contain different cell subtypes that obey either the SPM or the classical CSC model (since both models, and the other newly proposed alternative models, are not necessarily mutually exclusive), the ultimate goal should be to eradicate both CSCs and non-CSCs at once and, accordingly, to design novel clinical trials in a more coherent manner [85]. 

## 4. Conclusions

As more studies are being published regarding isolation and characterization of CSCs in different cancer types, it is becoming more evident that the so far established stem cells model cannot be universally applied as previously described for gliomas [6], melanomas [91], and hepatocellular carcinomas [64]. Our paper provides further support for alternative models of cancer biology that favour the existence of a single type of cancer cell with different stemness properties highly dependent on the microenvironmental conditions. The SPM originally proposed for gliomas [6] is an attractive and simple working model that reconciles experimental data with the stem cell hypothesis. Most of the key proposal or predictions of the SPM have been observed in melanomas (rare subpopulations of slow dividing cells, interconversion, and modulation of stemness by external factors) and other solid tumors briefly discussed in specific sections of this paper. The accumulative experimental evidence provides a strong indication that the SPM can be extrapolated to other types of cancer. 

## Figures and Tables

**Table 1:** Detection of CSCs in established breast cancer cell lines.

Cell line	Percentage	Method	Reference
BT-474	0.5%	SP (H33342 labeling)	[16]
EFM-19	0.3%	SP (H33342 labeling)	[16]
KPL-1	8.4%	SP (H33342 labeling)	[16]
MCF-7	0.8%	SP (H33342 labeling)	[16]
UACC-893	20%	SP (H33342 labeling)	[16]
BT-20	5.8%	SP (H33342 labeling)	[16]
Cal-51	0.8–2%	SP (H33342 labeling)	[16]
MCF-7	2.4 ± 0.4%	SP (H33342 labeling)	[17]
MCF-7	1.5%	SP (H33342 labeling)	[18]
T47D,	0%	SP (H33342 labeling)	[17]
SK-BR-3	0%	SP (H33342 labeling)	[17]
MDA-MB-231	0%	SP (H33342 labeling)	[17]
MDA-MB-231	0.1%	SP (H33342 labeling)	[18]
MDA-MB-231	>90%	CD44^pos^/CD24^neg/low^ (flow cytometry)	[19]
MCF-7	1.6% (monolayer cultures) 40.3% (mammospheres)	CD44^pos^/CD24^neg/low^ (flow cytometry)	[20]
MCF-7	<2% (monolayer cultures) ~50% (mammospheres)	CD44^pos^/CD24^neg/low^ (flow cytometry)	[21]
MCF-7	3.5%	SP (H33342 labeling)	[22]

**Table 2:** Detection of CSCs in established lung cancer cell lines.

Cell line	Percentage	Method	Reference
LHK2, 1-87, A549, Lc817	0.4% to 2.8%	SP	[23]
A549	0.98%	CD133	[24]
H446	1%	CD133	[24]
A549	>45%	Cloning and tumorigenic analyses	[25]
H446	>45%	Cloning and tumorigenic analyses	[25]
A549	24%	SP	[26]
H460, H23, HTB-58, A549, H441, and H2170	1.5% to 6.1%	SP	[27]
NCI-H82, H146, H526, A549, and H460	0.8% to 1%	SP	[28]
H446	6.3 ± 0.1	SP	[29]
NSCLC cell lines H460, H125, H322, H358	average of 2% (2.16 ± 1.28)	Aldefluor followed by clonogenic assays	[30]
A549, H1299, CCL-1, CCL-5, C299	0.3% to 1%	CD133^+^ follow serum free culture	[31]
60 primary tissue samples	0.02% to a maximum of 35%	CD133^+^ESA^+^	[32]

**Table 3:** Detection of CSCs in established prostate cancer cell lines.

Cell line	Percentage	Method	Reference
Human prostate basal cell isolation (primary culture)	1%	CD133^+^ cells	[33]
LNCaP	0.04%	CD44^+^ CD24^-^	[34]
DU145	7–10%	CD44^+^ CD24^-^	[34]
Dul45	50.67 ± 6.7	CD44^+^ cells	[35]
LAPC-4	0.847 ± 0.3	CD44^+^ cells	[35]
LAPC-9	13.97 ± 3.2	CD44^+^ cells	[35]
Primary cultures from 5 different patients	0.3–1.6	CD44^+^/*α*2*β*1^hi^/CD133^+ ^Cells	[36]
PC-3, PC3P, PC3MM2	0.2, 0.6, 0.7	CD133^high^	[37]

**Table 4:** Detection of CSCs in established melanoma cell lines.

Cell line	Percentage	Method	Reference
B16F10	<3.4%	CD44+CD133+CD24+ (flow cytometry)	[65]
WM115	0.71%	CD133+ (flow cytometry)	[66]
Cell suspension from biopsies (7 specimens)	<1%	CD133+ (flow cytometry)	[66]
Cell suspension from biopsies (7 specimens)	1.6 to 20.4%	ABCB5 (flow cytometry)	[67]
WM-266-4	Small (not quantified)	Spheroids	[68]

**Table 5:** External (microenvironmental) modulators of stemness.

Factor	Cell line (cancer)	Effect on stemness	Reference
Extracellular ATP	Gliomas	↓	[69]
High energy metabolites (lactate and ketones)	MCF-7 (breast)	↑	[70]
Hypoxia and the hypoxic microenvironment	Prostate, brain, kidney, cervix, lung, colon, liver, and breast tumors	↑	[71–74]
Hepatocyte growth factor	Colon	↑	[75]
VEGF	Skin	↑	[76]
VEGF	Gliomas	↑	[77]
Nitric oxide	Gliomas	↑	[78]
Retinoic acid	Gliomas	↓	[79]
ROS	Breast	↑	[80]
Conditioned medium	DU145	↑ or ↓ depending on the media	[81]

	Non-cancer		

Hypoxia	Embryonic stem cells	↑	[82]
Hypoxia	Human embryonic stem (hES) cells	↑	[83]

**Table 6:** Comparison between the SPM and other alternative models.

Model	Hierarchy	Interconversion between CSCs and non-CSCs	Influence of the microenvironment on stemness	Origin of heterogeneity	Reference
Classical CSC model	Yes	No	No^a^	Existence of different subpopulations. The origin of each subpopulation (e.g., CSCs) is controversial.	[6, 15, 84, 85]
SPM	No	Yes	Yes	Microenvironment-driven phenotypic changes	[6]
Complex system model	Partial (some differentiated cells may not interconvert)	Probably yes (not clearly mentioned)	Probably yes (referred at “the niche”)	Genetic, epigenetics Cell-cell and cell-niche interactions	[84]
Dynamic CSC model	No	Yes	Yes	Genetic, epigenetics, microenvironment-driven phenotypic changes	[85]
Reprogramming model	No	Yes	Yes	Epigenetics, endogenous transcriptional reprogramming networks, microenvironmental signals	[15]

^a^In the classical CSC, cells are preferentially enriched in specific niches rather than shaped by the microenvironment.
